# Use of liquid platelet‐rich fibrin (liquid PRF) in temporomandibular joint disorders: A narrative review with clinical recommendations

**DOI:** 10.1111/prd.70012

**Published:** 2025-09-26

**Authors:** Nima Farshidfar, Mohammad Amin Amiri, Nathan E. Estrin, Paras Ahmad, Yufeng Zhang, Anton Sculean, Richard J. Miron

**Affiliations:** ^1^ Department of Periodontology University of Bern Bern Switzerland; ^2^ Department of Research Advanced PRF Education Jupiter Florida USA; ^3^ Lake Erie College of Osteopathic Medicine School of Dental Medicine Bradenton Florida USA; ^4^ Department of Oral Implantology University of Wuhan Wuhan China

**Keywords:** concentrated PRF, C‐PRF, injectable platelet‐rich fibrin, i‐PRF, temporomandibular joint

## Abstract

**Aim:**

This narrative review aimed to gather evidence from comparative and non‐comparative clinical studies to assess: (1) whether the administration of liquid platelet‐rich fibrin (liquid PRF) provides any clinical benefits for managing temporomandibular disorders (TMD), and if so, based on comparative clinical studies, (2) whether it offers more benefits than no treatment or other treatment modalities, either as a standalone therapy or as an adjunct.

**Materials and Methods:**

To compile all relevant data, we performed a systematic search of PubMed, Scopus, and Web of Science, supplemented by a Google Scholar search for gray literature and a manual screening of reference lists from eligible studies and relevant reviews, up to April 22, 2025. A total of 23 clinical studies (19 comparative and 4 non‐comparative) were ultimately included in this review.

**Results:**

Across the included studies, the administration of liquid PRF has been shown to have beneficial effects in reducing pain and enhancing maximum mouth opening (MMO) in patients with TMD. In the majority of studies, the adjunctive use of liquid PRF following arthrocentesis demonstrated greater positive effects compared with arthrocentesis alone. When used adjunctively with arthrocentesis, liquid PRF also showed comparable or superior clinical outcomes in terms of pain reduction and MMO improvement compared with platelet‐rich plasma (PRP) and hyaluronic acid (HA).

**Conclusions:**

Based on these findings, the administration of liquid PRF following arthrocentesis appears to be a promising approach for the management of TMD. To support clinical application, this review also presented a step‐by‐step protocol to guide dental and medical practitioners in the effective use of liquid PRF in patients with TMD. However, further well‐designed randomized clinical trials with standardized methodologies are required to strengthen the evidence base and confirm the therapeutic benefits of liquid PRF in the management of TMD due to the high variability among the included studies.

## 1 INTRODUCTION

Temporomandibular joint disorders (TMD) are a prevalent and multifactorial subset of craniofacial pain conditions that affect the temporomandibular joint (TMJ), the masticatory muscles, and related musculoskeletal structures of the head and neck.^1^, ^2^, ^3^ Patients with TMD commonly present with symptoms such as pain, restricted or uneven mandibular movement, and joint sounds.^2^, ^4^ Treatment options for TMD range from noninvasive approaches to surgical interventions, all of which aim to alleviate pain, restore normal mandibular movement, and improve quality of life.^5^, ^6^, ^7^ Noninvasive treatments, typically including pharmacotherapy, physiotherapy, behavioral therapy, and occlusal splints, are considered first‐line management. However, due to their limited efficacy, they are often followed by minimally invasive approaches, which offer more safety and lower morbidity compared with invasive surgical interventions.^8^ Among these, arthrocentesis is a widely accepted, minimally invasive procedure that involves irrigating the joint with saline or Ringer's solution to reduce intra‐articular pressure and remove inflammatory mediators.^9^, ^10^

**FIGURE 1:**
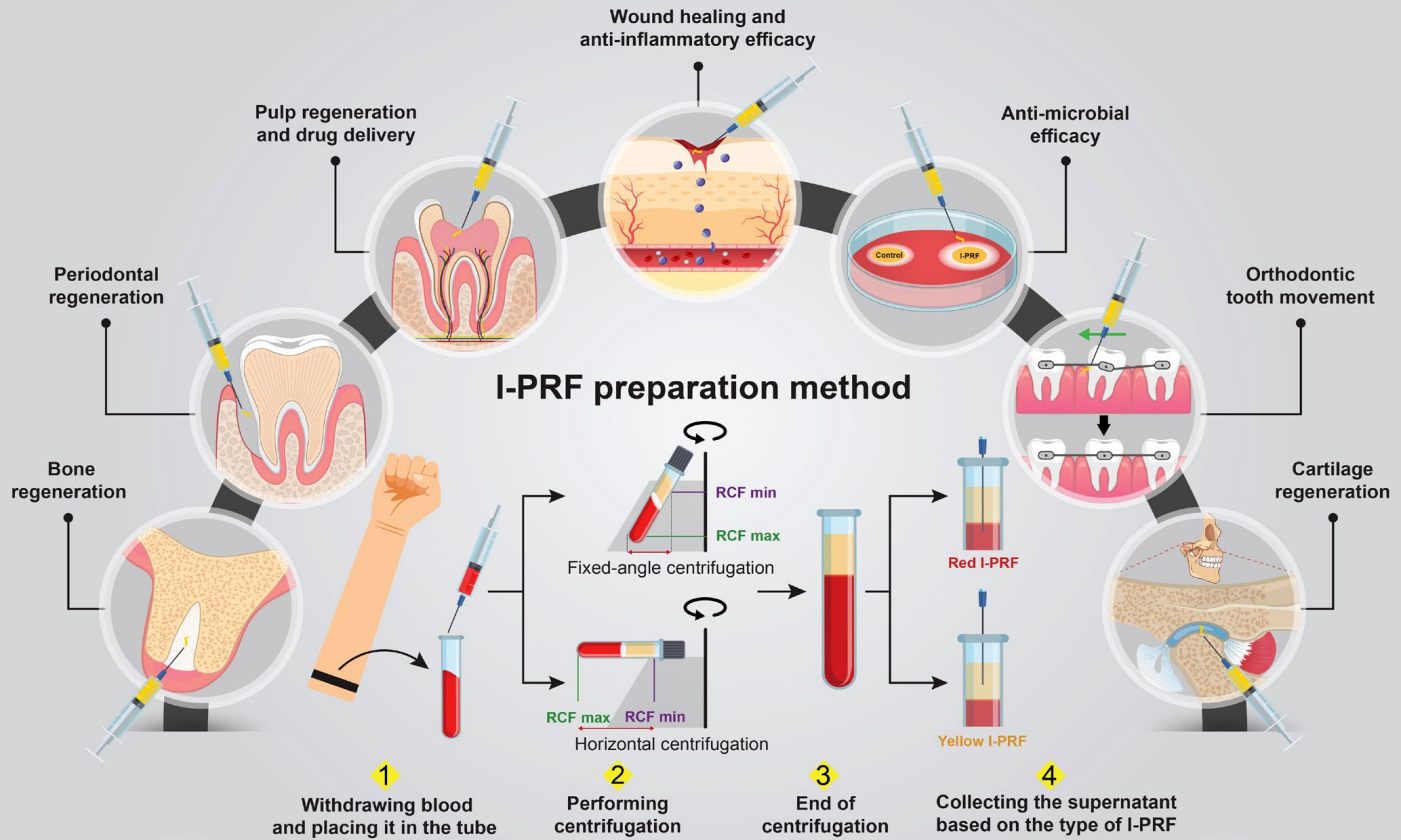
Schematic illustration depicting the regenerative capacities of liquid PRF in relation to oral and maxillofacial structures, along with its preparation method. Reprinted with permission from Farshidfar et al.^25^

Recently, the use of injectable therapeutic agents, such as corticosteroids, hyaluronic acid (HA), and autologous platelet concentrates (APCs) has gained increasing attention for their potential to improve outcomes of TMD treatment, either as standalone therapies or as adjuncts to procedures such as arthrocentesis.^11^, ^12^ Specifically, APCs have been widely utilized in dentistry and medicine owing to their regenerative potential and serving as all‐natural biomaterials.^13^, ^14^, ^15^, ^16^, ^17^, ^18^, ^19^, ^20^, ^21^, ^22^, ^23^ Since 2015, a liquid version of PRF (liquid PRF or i‐PRF) has been developed as an injectable second‐generation APC, with promising regenerative capabilities (Figure 1).^24^, ^25^, ^26^, ^27^ A recent systematic review investigating studies across all fields of medicine and dentistry found that 72% of studies favored the use of liquid PRF when compared with platelet‐rich plasma (PRP), whereas 24% of studies demonstrated no difference.^28^ Thus, to date, strong evidence exists for the use of liquid PRF versus PRP for the management of TMD.

Following application, the fibrinogen in liquid PRF is gradually transformed into a fibrin clot (PRF), which releases growth factors continuously over 10–14 days.^26^, ^29^, ^30^ This sustained release creates a favorable microenvironment that promotes cellular recruitment, proliferation, and differentiation, all of which are critical for tissue regeneration.^31^, ^32^ In addition to its regenerative potential, PRF has also demonstrated notable anti‐inflammatory properties, which can help modulate the inflammatory response and further enhance the regeneration and healing process.^32^, ^33^, ^34^ This highlights the potential of liquid PRF, due to its regenerative and anti‐inflammatory/antimicrobial benefits, as a treatment option for TMD that is able to enhance clinical outcomes.^35^

There are a few published review articles that have evaluated the efficacy of liquid PRF administration in patients with TMD;^8^, ^36^, ^37^, ^38^ however, none have offered a comprehensive assessment or mapped the current literature. Accordingly, this narrative review aimed to provide the most up‐to‐date comprehensive overview of the current literature assessing the efficacy of liquid PRF, either as a standalone therapy or as an adjunct to other treatment modalities. By analyzing evidence from comparative and non‐comparative clinical studies, this review sought to clarify: (1) whether the administration of liquid PRF offers any clinical benefits, and if so, based on comparative clinical studies, (2) whether it offers more benefits than no treatment or other treatment modalities, either as a standalone therapy or as an adjunct. In addition, limitations and future prospects were discussed, and based on the available evidence, a step‐by‐step protocol was provided to guide dental and medical practitioners toward the effective use of liquid PRF for patients with TMD.

## 2 SEARCH STRATEGY

To ensure compilation of all relevant data from the literature, two authors (N.F. and MA.A.) independently conducted a systematic search of the PubMed, Scopus, and Web of Science databases using the keywords listed in Table 1, applied to the titles and abstracts of published studies. Articles published up to April 22, 2025, were considered. Additionally, the same authors also independently screened gray literature (i.e., Google Scholar) using a combination of keywords listed in Table 1 and reviewed the reference lists of eligible studies and relevant review articles to identify any additional pertinent publications. Eligibility criteria for study inclusion and exclusion were established using the Participants, Intervention, Comparison, Outcome, and Study Design (PICOS) framework (Table 2). Studies not published in English were excluded to maintain consistency and ensure accurate interpretation. Pain and maximum mouth opening (MMO) were used as clinical outcome measures to assess whether liquid PRF, either as a standalone therapy or as an adjunct to other treatment modalities, offers any clinical benefits.

**TABLE 1:** Strategies for main database searches up to April 22, 2025.

Database	Search Strategy	Hits
PubMed	((Temporo[Title/Abstract]) OR (Temporomandibular[Title/Abstract]) OR (Temporomandibular Joint[Title/Abstract]) OR (TMJ[Title/Abstract]) OR (Temporomandibular Disc[Title/Abstract]) OR (Temporomandibular Joint Disc[Title/Abstract]) OR (Temporomandibular Disorder[Title/Abstract]) OR (TMD[Title/Abstract]) OR (Temporomandibular Joint Disorder[Title/Abstract]) OR (Temporomandibular dysfunction[Title/Abstract])) AND ((Platelet rich fibrin[Title/Abstract]) OR (Platelet‐rich Fibrin[Title/Abstract]) OR (PRF[Title/Abstract]) OR (injectable Platelet Rich Fibrin[Title/Abstract]) OR (Injectable Platelet‐rich Fibrin[Title/Abstract]) OR (i‐PRF[Title/Abstract]) OR (Liquid Platelet Rich Fibrin[Title/Abstract]) OR (Liquid Platelet‐rich Fibrin[Title/Abstract]) OR (Liquid PRF[Title/Abstract]))	38
Scopus	(TITLE‐ABS‐KEY (“Temporo” OR “Temporomandibular” OR “Temporomandibular Joint” OR “TMJ” OR “Temporomandibular Disc” OR “Temporomandibular Joint Disc” OR “Temporomandibular Disorder” OR “TMD” OR “Temporomandibular Joint Disorder” OR “Temporomandibular dysfunction”) AND TITLE‐ABS‐KEY (“Platelet rich fibrin” OR “Platelet‐rich Fibrin” OR “PRF” OR “injectable Platelet Rich Fibrin” OR “Injectable Platelet‐rich Fibrin” OR “i‐PRF” OR “Liquid Platelet Rich Fibrin” OR “Liquid Platelet‐rich Fibrin” OR “Liquid PRF”))	53
Web of Science	“Temporo” OR “Temporomandibular” OR “Temporomandibular Joint” OR “TMJ” OR “Temporomandibular Disc” OR “Temporomandibular Joint Disc” OR “Temporomandibular Disorder” OR “TMD” OR “Temporomandibular Joint Disorder” OR “Temporomandibular dysfunction” (Topic) and “Platelet rich fibrin” OR “Platelet‐rich Fibrin” OR “PRF” OR “injectable Platelet Rich Fibrin” OR “Injectable Platelet‐rich Fibrin” OR “i‐PRF” OR “Liquid Platelet Rich Fibrin” OR “Liquid Platelet‐rich Fibrin” OR “Liquid PRF” (Topic)	45

**TABLE 2:** Eligibility criteria for the present review.

Domains	Inclusion Criteria	Exclusion Criteria
Participants	Studies involving patients with TMD	Studies involving cells Studies involving animal models
Intervention	Injection of liquid PRF, either as a standalone treatment or as an adjunct to other treatment modalities	–
Comparison	No control No treatment Injection of alternative therapeutic agents	–
Outcome	TMJ pain and maximum mouth opening (MMO)	–
Study design	Clinical comparative and non‐comparative studies	Case series Case reports Preclinical studies (in vitro and in vivo studies) Review articles Conference proceedings Protocol articles Letters to the editor Book chapters

## 3 RESULTS

**FIGURE 2:**
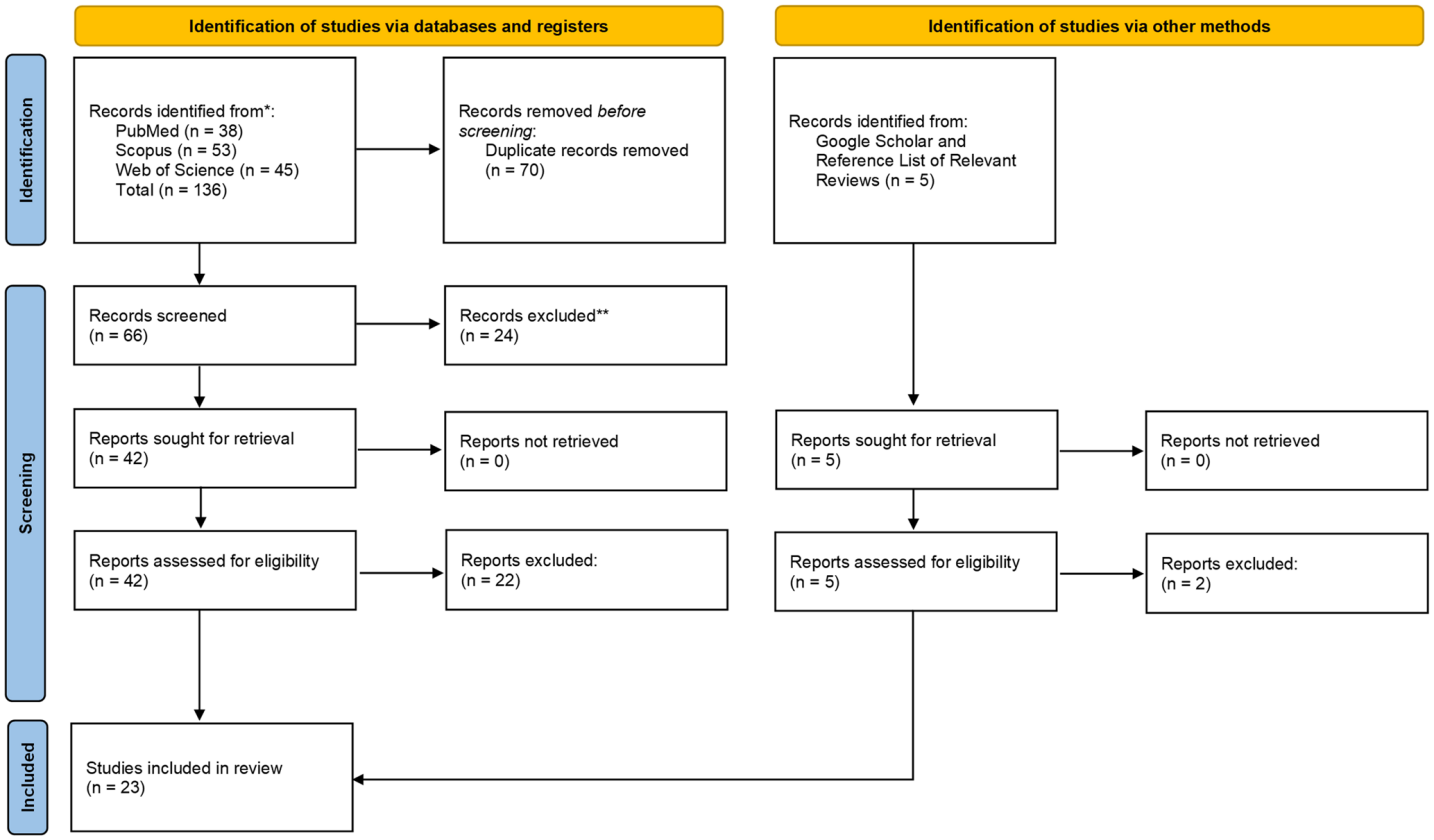
Flowchart illustrating the study selection process.

In total, 23 clinical studies (19 comparative studies and 4 non‐comparative) were ultimately selected for inclusion in our review, comprising 20 from the primary databases and three additional studies identified through gray literature and reference list screening of relevant reviews — Figure 2 shows the study selection process in detail.

Tables 3 and 4 provided a detailed summary of the studies included. In brief, a total of 1,316 patients (163 males, 852 females, and 301 unspecified) were included in this review. The types of TMD ranged from internal derangements to non‐inflammatory degenerative joint disorders and TMJ hypermobility, except for two studies that did not specify the type of TMD. Liquid PRF was administered either as a standalone treatment or as an adjunct to arthrocentesis. Most studies used a centrifugation protocol of 700 rpm for 3 min to prepare liquid PRF, except for three studies — one did not specify the protocol, while the other two used 3300 rpm for 2 min and 630 rpm for 3 min. The most common injection site in TMD patients was the superior joint space, although other sites were also reported.

**TABLE 3:** Detailed summary of the included comparative clinical studies.

Author (Year)	Study design	Type of TMD (subtype)	Sample size (M/F)	Study Groups (amount of injection or name of the solution) × number of injection	Number of Treated patients (number of treated sites)	Liquid PRF preparation method (device)	Injection site	Main measured parameters (measurement tool)	Time points assessment	Main outcomes
Retrospective clinical studies										
Tepecik et al. (2025) [39]	Retrospective Clinical Study	Non‐inflammatory degenerative joint disorder (Osteoarthritis)	85 (0/85)	I: Arthrocentesis (Saline solution) × 1 + Liquid PRF (1.5 mL) × 1 II: Arthrocentesis × 1 + Liquid PRF (1.5 mL) × 3* *Every week	I: 45 (45) II: 40 (40)	700 rpm 3 min (NR)	Superior joint space	(1) Pain (VAS) (2) MMO (NR)	1, 6, and 12 months	No significant differences were observed between the two groups in terms of pain reduction and improvement in MMO
Tepecik et al. (2025) [40]	Retrospective Clinical Study	Non‐inflammatory degenerative joint disorder (Osteoarthritis)	127 (0/127)	I: Arthrocentesis (Saline solution) × 1 II: Arthrocentesis × 1 + HA (1.5 mL) × 1 III: Arthrocentesis × 1 + Liquid PRF (1.5 mL) × 1	I: 45 (45) II: 39 (39) III: 43 (43)	700 rpm 3 min (NR)	Superior joint space	(1) Pain (VAS) (2) MMO (NR)	1, 6, and 12 months	Both liquid PRF and HA groups resulted in significantly greater pain relief than arthrocentesis alone, with no significant difference observed between them. MMO showed no significant differences among the three groups
Akkas et al. (2024) [41]	Retrospective Clinical Study	NR	116 (13/103)	I: Arthrocentesis (Saline solution) × 1 II: Arthrocentesis × 1 + PRP (2 mL) × 1 III: Arthrocentesis × 1 + Liquid PRF (2 mL) × 1	I: 35 (35) II: 34 (34) III: 47 (47)	700 rpm 3 min (NR)	Superior joint space	(1) Pain (VAS) (2) MMO (Analog Caliper)	1 week, 1,3, and 6 months	No significant difference in pain was found among the groups. However, PRP showed better early postoperative mouth opening, while liquid PRF showed better outcomes later
Tepecik et al. (2024) [42]	Retrospective Clinical Study	Internal derangement disorder (Disc displacement without reduction)	88 (0/88)	I: Arthrocentesis (Saline solution) × 1 II: Arthrocentesis × 1 + HA (1.5 mL) × 1 III: Arthrocentesis + Liquid PRF (1.5 mL) × 1	I: 30 (30) II: 29 (29) III: 29 (29)	700 rpm 3 min (NR)	Superior joint space	(1) Pain (VAS) (2) MMO (Caliper)	1 and 6 months	At 6 months, pain scores in the liquid PRF and HA groups were similar and significantly better than those in the arthrocentesis group. No differences were observed in MMO between the groups
Bera et al. (2022) [43]	Retrospective Clinical Study	Non‐inflammatory degenerative joint disorder (Osteoarthritis)	130 (NR)	I: Arthrocentesis (Ringer's lactate solution) × 6* II: Arthrocentesis × 6* + Liquid PRF (2 mL) × 6* *Every 15 days	I: 67 (NR) II: 63 (NR)	700 rpm 3 min (NR)	Superior joint space	(1) Pain (VAS)	15 days, 1, 3, and 6 months	There was no statistical difference in mean pain scores at the end of 6 months. MMO was significantly greater in the liquid PRF group at 12 months than arthrocentesis group
								(2) MMO (NR)	1, 3, 6, and 12 months	
Yuce et al. (2020) [44]	Retrospective Clinical Study	Internal derangement disorder (NR)	47 (23/24)	I: Arthrocentesis (Ringer's lactate solution) × 1 II: Arthrocentesis × 1 + HA (1 mL) × 1 III: Arthrocentesis × 1 + Liquid PRF (2 mL) × 3* * Every week	I: 16 (22) II: 14 (21) III: 17 (24)	700 rpm (60 g) 3 min (NR)	Superior joint space	(1) Pain (VAS) (2) MMO (Digital Caliper)	2 weeks, 1,2,3,6,9, and 12 months	The liquid PRF group showed significantly lower pain levels than the HA group at 9 months MMO was also significantly higher in the liquid PRF group compared with the HA group at both 9 and 12 months
Prospective clinical studies										
Vingender et al. (2023) [45]	Prospective Clinical Study	Internal derangement disorder (NR)	68 (9/59)	I: HA (1 mL) × 3* II: PRP (NR) × 1 III: Liquid PRF (NR) × 1 * Every week	I: 28 (NR) II: 21 (NR) III: 19 (NR)	NR	Superior joint space	(1) Pain (VAS) (2) MMO (NR)	6 and 12 months	No significant differences in pain reduction were observed among the groups. Only the HA group showed a significant increase in MMO at both time points
Randomized clinical trials										
Chaulagain et al. (2025) [46]	Randomized Clinical Trial (Parallel)	Internal derangement disorder (NR)	48 (12/36)	I: Arthrocentesis (Ringer's lactate solution) × 1 II: Liquid PRF (2–3 mL) × 1 III: Arthrocentesis × 1 + Liquid PRF (2–3 mL) × 1	I: 16 (16) II: 16 (16) III: 16 (16)	700 rpm 3 min (Choukron's PRF Centrifuge, NR)	Joint space	(1) Pain (VAS) (2) MMO (Calibrated scale)	10 days, 1, and 3 months	The arthrocentesis + liquid PRF group showed a significantly greater reduction in pain compared with the other two groups. This group also demonstrated significant improvements in MMO compared with the arthrocentesis‐only and liquid PRF‐only groups
Kumar et al. (2024) [47]	Randomized Clinical Trial (Parallel)	NR	68 (17/51)	I: No treatment II: Liquid PRF (1 mL) × 2* * Every week	I: 34 (NR) II:34 (NR)	700 rpm 3 min (Hettich EBA 20 centrifuge, Germany)	Superior joint space	(1) Pain (VAS) (2) MMO (NR)	6, 12, and 24 weeks	Liquid PRF demonstrated significant efficacy in reducing TMJ pain intensity and improving jaw function
Nasef et al. (2024) [48]	Randomized Clinical Trial (Parallel)	Internal derangement disorder (Bilateral Disc displacement without reduction)	30 (NR)	I: Anterior Repositing Splint + Arthrocentesis (Ringer's lactate solution) × 1 II: Anterior Repositing Splint + Arthrocentesis × 1 + Liquid PRF (NR) × 1	I: 15 (30) II: 15 (30)	700 rpm 3 min (NR)	Superior joint space	(1) Pain (VAS) (2) MMO (NR)	1 week, 1, 3, and 6 months	The liquid PRF group demonstrated significantly superior outcomes in reducing pain and improving MMO compared with arthrocentesis alone
Abdelrahman et al. (2024) [49]	Randomized Clinical Trial (Parallel)	Internal derangement disorder (NR)	30 (4/26)	I: Dextrose (3 mL) × 2* II: Liquid PRF (3 mL) × 1 * 4 weeks	I: 15 (NR) II:15 (NR)	700 rpm 3 min (NR)	Posterior joint space, anterior disc attachment to the lateral pterygoid muscle, and masseter attachment	(1) Pain (VAS) (2) MMO (NR)	1 week, 1, 3, and 6 months	Liquid PRF resulted in significantly lower pain levels at all time points and improved mouth opening at 1, 3, and 6 months
Isik et al. (2023) [50]	Randomized Clinical Trial (Parallel)	Internal derangement disorder (Disc displacement without reduction)	76 (7/69)	I: Arthrocentesis (Saline solution) × 1 II: Arthrocentesis × 1 + Liquid PRF (1 mL) × 4* * Every week	I: 38 (44) II: 38 (43)	700 rpm (60 g) 3 min (NR)	Superior joint space	(1) Pain (VAS) (2) MMO (Ruler)	1, 2, 3, 6, and 12 months	Liquid PRF group led to significantly greater reductions in pain and more notable enhancements in MMO compared with the control group
Sharma et al. (2023) [51]	Randomized Clinical Trial (Parallel)	Internal derangement disorder (NR)	14 (NR)	I: Arthrocentesis (Ringer's lactate solution) × 1 + PRP (2 mL) × 6* II: Arthrocentesis × 1 + Liquid PRF (2 mL) × 6* * Every month	I: 7 (14) II: 7 (14)	700 rpm 3 min (NR)	Superior joint space	(1) Pain (VAS) (2) MMO (More Rao Scale)	1, 2, 3, 4, 5, 6, and 9 months	The liquid PRF group showed greater improvements across clinical parameters compared with the PRP group
Isik et al. (2022) [52]	Randomized Clinical Trial (Parallel)	Non‐inflammatory degenerative joint disorder (Osteoarthritis)	36 (3/33)	I: Arthrocentesis (Saline solution) × 1 II: Arthrocentesis × 1 + Liquid PRF (1 mL) × 4* * Every week	I: 18 (21) II: 18 (22)	700 rpm (60 g) 3 min (NR)	Superior joint space	(1) Pain (VAS) (2) MMO (Ruler)	1, 2, 3, 6, and 12 months	Liquid PRF demonstrated significantly greater improvements in pain reduction and MMO compared with the control group
Ghoneim et al. (2021) [53]	Randomized Clinical Trial (Parallel)	Internal derangement disorder (Disc displacement with reduction)	40 (11/29)	I: Arthrocentesis (Ringer's lactate solution) × 1 II: Arthrocentesis × 1 + Liquid PRF (1.5 mL) × 1	I: 20 (NR) II: 20 (NR)	700 rpm (60 g) 3 min (Spin plus Centrifuge, China)	Superior joint space	(1) Pain (VAS) (2) MMO (Caliper)	1 week, 3, and 6 months	The liquid PRF group showed a statistically significant reduction in pain intensity along with an increase in MMO, compared with the arthrocentesis group
Karadayi et al. (2021) [54]	Randomized Clinical Trial (Parallel)	Internal derangement disorder (NR)	36 (19/17)	I: Arthrocentesis (Ringer's lactate solution) × 1 II: Arthrocentesis × 1 + Liquid PRF (2 mL) × 1	I: 18 (18) II: 18 (18)	700 rpm 3 min (NR)	Superior joint space	(1) Pain (VAS) (2) MMO (Caliper)	10 and 30 days, and 3 months	The liquid PRF group showed significantly greater VAS improvements. It also significantly improved MMO only at the second time point; changes at the first and third were not significant
Torul et al. (2021) [55]	Randomized Clinical Trial (Parallel)	Internal derangement disorder	54 (2/52)	I: Arthrocentesis (Ringer's lactate solution) × 1 II: Arthrocentesis × 1 + HA (1 mL) × 1 III: Arthrocentesis × 1 + Liquid PRF (1 mL) × 1	I: 18 (NR) II: 18 (NR) III: 18 (NR)	700 rpm (60 g) 3 min (Duo Centrifuge, France)	NR	(1) Pain at rest and function (VAS) (2) MMO (Digital Caliper)	1 week, 1, and 3 months	Compared with the HA and arthrocentesis groups, the liquid PRF group demonstrated greater improvements in MMO and greater reductions in pain intensity at rest (1 and 3 months) and during function (1 week, 1 month, and 3 months)
Teama et al. (2020) [56]	Randomized Clinical Trial (NR)	Internal derangement disorder (NR)	30 (NR)	I: Superior Repositioning Splint + Arthrocentesis (Saline Solution) × 1 II: Superior Repositioning Splint + Arthrocentesis × 1 + L‐PRF (1.5–2 mL) × 1	I: NR (18) II: NR (18)	3300 rpm 2 min (NR)	Superior joint space	(1) Pain (VAS) (2) MMO (Caliper)	1, 2, 4, and 8 weeks	The liquid PRF group outperformed the standard arthrocentesis group in pain relief, speed, and extent of MMO recovery
Baiomy et al. (2019) [57]	Randomized Clinical Trial (Parallel)	TMJ hypermobility (Dislocation)	30 (NR)	I: Autologous blood (5 mL) × 1 II: Liquid PRF (5 mL) × 1	I: 15 (NR) II: 15 (NR)	700 rpm (60 g) 3 min (NR)	Joint space and pericapsular tissue	(1) Pain (VAS) (2) MMO (NR)	1 and 3 months	The liquid PRF group showed significant improvements in both pain and MMO, compared with the autologous blood group

*Note*: The type of TMD was classified according to the classification described by Scrivani et al.^58^

Abbreviations: HA, hyaluronic acid; M/F, male/female; min, minute; mL, milliliter; MMO, maximum mouth opening; NR, not reported; PRF, platelet‐rich fibrin; PRP, platelet‐rich plasma; rpm, revolutions per minute; TMD, temporomandibular disorders; TMJ, temporomandibular joint; VAS, visual analog scale.

**TABLE 4:** Detailed summary of the included non‐comparative clinical studies.

Author (year)	Study design	Type of TMD (subtype)	Sample size (M/F)	Study groups (amount of injection or name of the solution) × number of injection	Number of treated patients (number of treated sites)	Liquid PRF preparation method (device)	Injection site	Main measured parameters (measurement tool)	Time points assessment	Main outcomes
Prospective clinical studies										
Sielski et al. (2025) [59]	Prospective Clinical Study	Internal derangement disorder (NR)	33 (25/8)	I: Liquid PRF (0.4 mL) × 1	I: 33 (44)	630 rpm 3 min (D06 Centrifuge, India)	Superior joint space	(1) Pain (VAS) (3) MMO (NR)	2 weeks	TMJ pain showed significant reduction, whereas changes in masticatory muscle, head, and neck pain were not significant; however, MMO showed a significant decrease
Aishwarya et al. (2024) [60]	Prospective Clinical Study	TMJ hypermobility (Dislocation and Subluxation)	26 (12/14)	I: Liquid PRF (4 mL) × 1	I: 26 (NR)	700 rpm (60 g) 3 min (NR)	Superior joint space and pericapsular area	(1) Pain (VAS) (2) MMO (Ruler)	1 and 3 months	The MMO and pain scores showed significant reductions from baseline to the 3‐month follow‐up
Shukla et al. (2024) [61]	Prospective Clinical Study	Internal derangement disorder (NR)	67 (NR)	I: Liquid PRF (1.5–2 mL) × 1	I: 67 (NR)	700 rpm 3 min (Duo Centrifuge, France)	Superior joint space	(1) Pain (VAS)	15 days, 1, 3, and 6 months	VAS scores significantly decreased at each follow‐up interval. At 6 months, over 75% of cases had scores reduced to 0, and 91.5% had scores of 2 or less
Albilia et al. (2020) [62]	Prospective Clinical Study	Internal derangement disorder (NR)	37 (6/31)	I: Liquid PRF (2 mL) × 2* * Every 2 weeks	37 (48)	700 rpm (60 g) 3 min (Duo Centrifuge, France)	Superior joint space and retrodiscal tissue	(1) Pain (VAS) (2) MMO (NR)	2 weeks, 1, 2, 3, 6, 9, and 12 months	No statistically significant improvement was found in pain or MMO, though all showed favorable trends

*Note*: The type of TMD was classified according to the classification described by Scrivani et al.^58^

Abbreviations: M/F, male/female; min, minute; mL, milliliter; MMO, maximum mouth opening; PRF, platelet‐rich fibrin; rpm, revolutions per minute; TMJ, temporomandibular joint; TMD, temporomandibular disorders; VAS, visual analog scale; NR, not reported.

### 3.1 Clinically relevant questions

Based on the included studies, we aimed to answer the following clinically relevant questions using the evidence gathered from the literature:

*#1 Does the sole administration of liquid PRF offer any benefit in reducing pain and improving MMO in patients with TMD, based on comparisons between baseline and follow‐up measurements?*

100% of the findings from the included studies in this section showed that the sole administration of liquid PRF had positive effects in reducing pain, and 87.5% in improving MMO. Only one finding (12.5%) reported no benefits of the sole use of liquid PRF for improving MMO (Tables 5 and 6).

**TABLE 5:** Summary of findings from studies evaluating whether the sole administration of liquid PRF provides any benefits in reducing pain in patients with TMD, based on comparisons between baseline and follow‐up measurements.

Authors (years)	Type of study	Experimental group (amount of injection) × Number of injection	Did the treatment provide any benefit?
Non‐comparative clinical studies			
Sielski et al. (2025) [59]	Prospective clinical study	Liquid PRF (0.4 mL) × 1	Yes
Aishwarya et al. (2024) [60]	Prospective Clinical Study	Liquid PRF (4 mL) × 1	Yes
Shukla et al. (2024) [61]	Prospective Clinical Study	Liquid PRF (1.5–2 mL) × 1	Yes
Albilia et al. (2020) [62]	Prospective Clinical Study	Liquid PRF (2 mL) × 2	Yes
Comparative clinical studies			
Vingender et al. (2023) [45]	Prospective Clinical Study	Liquid PRF (−) × 1	Yes
Chaulagain et al. (2025) [46]	Randomized Clinical Trial	Liquid PRF (2–3 mL) × 1	Yes
Kumar et al. (2024) [47]	Randomized Clinical Trial	Liquid PRF (1 mL) × 2	Yes
Abdelrahman et al. (2024) [49]	Randomized Clinical Trial	Liquid PRF (3 mL) × 1	Yes
Baiomy et al. (2019) [57]	Randomized Clinical Trial	Liquid PRF (5 mL) × 1	Yes

*Note*: The color shading in the table indicates yes versus no.

**TABLE 6:** Summary of findings from studies evaluating whether the sole administration of liquid PRF provides any benefits in improving MMO in patients with TMD, based on comparisons between baseline and follow‐up measurements.

Authors (years)	Type of study	Experimental group (amount of Injection) × Number of Injection	Did the treatment provide any benefit?
Non‐comparative clinical studies			
Sielski et al. (2025) [59]	Prospective Clinical Study	Liquid PRF (0.4 mL) × 1	No
Aishwarya et al. (2024) [60]	Prospective Clinical Study	Liquid PRF (4 mL) × 1	Yes
Albilia et al. (2020) [62]	Prospective Clinical Study	Liquid PRF (2 mL) × 2	Yes
Comparative clinical studies			
Vingender et al. (2023) [45]	Prospective Clinical Study	Liquid PRF (−) × 1	Yes
Chaulagain et al. (2025) [46]	Randomized Clinical Trial	Liquid PRF (2–3 mL) × 1	Yes
Kumar et al. (2024) [47]	Randomized Clinical Trial	Liquid PRF (1 mL) × 2	Yes
Abdelrahman et al. (2024) [49]	Randomized Clinical Trial	Liquid PRF (3 mL) × 1	Yes
Baiomy et al. (2019) [57]	Randomized Clinical Trial	Liquid PRF (5 mL) × 1	Yes

*Note*: The color shading in the table indicates yes versus no.

*#2 Does the sole administration of liquid PRF offer additional benefit than no treatment or other standalone treatment modalities in reducing pain and improving MMO in patients with TMD?*

The sole administration of liquid PRF provided greater benefits in reducing pain and improving MMO compared with no treatment, dextrose prolotherapy, and autologous blood, while showing no difference compared with PRP or arthrocentesis with Ringer's lactate solution. However, liquid PRF was less effective than HA in improving MMO, while both showed similar effects in reducing pain (Tables 7 and 8).

**TABLE 7:** Summary of findings from studies evaluating whether the sole administration of liquid PRF provides more benefits than no treatment or other standalone treatment modalities in reducing pain in patients with TMD.

Authors (years)	Type of study	Control group (amount of injection or name of the solution) × Number of injection	Experimental group (amount of injection) × Number of injection	Which treatment was more beneficial?
Comparative clinical studies				
Vingender et al. (2023) [45]	Prospective Clinical Study	HA (1 mL) × 3	Liquid PRF (−) × 1	No Difference
		PRP (−) × 1	Liquid PRF (−) × 1	No Difference
Chaulagain et al. (2025) [46]	Randomized Clinical Trial	Arthrocentesis (Ringer's lactate solution) × 1	Liquid PRF (2–3 mL) × 1	No Difference
Kumar et al. (2024) [47]	Randomized Clinical Trial	No treatment	Liquid PRF (1 mL) × 2	Liquid PRF
Abdelrahman et al. (2024) [49]	Randomized Clinical Trial	Dextrose (3 mL) × 2	Liquid PRF (3 mL) × 1	Liquid PRF
Baiomy et al. (2019) [57]	Randomized Clinical Trial	Autologous blood (5 mL) × 1	Liquid PRF (5 mL) × 1	Liquid PRF

*Note*: The color shading in the table indicates yes versus no.

**TABLE 8:** Summary of findings from studies evaluating whether the sole administration of liquid PRF provides more benefits than no treatment or other standalone treatment modalities in improving MMO in patients with TMD.

Authors (years)	Type of study	Control group (amount of injection or name of the solution) × Number of injection	Experimental group (amount of injection) × Number of injection	Which treatment was more beneficial?
Comparative clinical studies				
Vingender et al. (2023) [45]	Prospective Clinical Study	HA (1 mL) × 3	Liquid PRF (−) × 1	Other Group
		PRP (−) × 1	Liquid PRF (−) × 1	No Difference
Chaulagain et al. (2025) [46]	Randomized Clinical Trial	Arthrocentesis (Ringer's lactate solution) × 1	Liquid PRF (2–3 mL) × 1	No Difference
Kumar et al. (2024) [47]	Randomized Clinical Trial	No treatment	Liquid PRF (1 mL) × 2	Liquid PRF
Abdelrahman et al. (2024) [49]	Randomized Clinical Trial	Dextrose (3 mL) × 2	Liquid PRF (3 mL) × 1	Liquid PRF
Baiomy et al. (2019) [57]	Randomized Clinical Trial	Autologous blood (5 mL) × 1	Liquid PRF (5 mL) × 1	Liquid PRF

*Note*: The color shading in the table indicates yes versus no.

*#3 Does the adjunctive administration of liquid PRF to arthrocentesis offer any benefit in reducing pain and improving MMO in the same patients with TMD, based on comparisons between baseline and follow‐up measurements?*

100% of findings from the included studies in this section showed that the adjunctive administration of liquid PRF to arthrocentesis has positive effects in both reducing pain and improving MMO (Tables 9 and 10).

**TABLE 9:** Summary of findings from studies evaluating whether the adjunctive administration of liquid PRF to arthrocentesis provides any benefits in reducing pain in patients with TMD, based on comparisons between baseline and follow‐up measurements.

Authors (years)	Type of study	Experimental group (amount of injection) × Number of injection	Did the treatment provide any benefit?
Comparative clinical studies			
Tepecik et al. (2025) [40]	Retrospective Clinical Study	Arthrocentesis (Saline solution) × 1 + Liquid PRF (1.5 mL) × 1	Yes
Akkas et al. (2024) [41]	Retrospective Clinical Study	Arthrocentesis (Saline solution) × 1 + Liquid PRF (2 mL) × 1	Yes
Tepecik et al. (2024) [42]	Retrospective Clinical Study	Arthrocentesis (Saline solution) × 1 + Liquid PRF (1.5 mL) × 1	Yes
Bera et al. (2022) [43]	Retrospective Clinical Study	Arthrocentesis (Ringer's lactate solution) × 6 + Liquid PRF (2 mL) × 6	Yes
Yuce et al. (2020) [44]	Retrospective Clinical Study	Arthrocentesis (Ringer's lactate solution) × 1 + Liquid PRF (2 mL) × 3	Yes
Chaulagain et al. (2025) [46]	Randomized Clinical Trial	Arthrocentesis (Ringer's lactate solution) × 1 + Liquid PRF (2–3 mL) × 1	Yes
Nasef et al. (2024) [48]	Randomized Clinical Trial	Anterior Repositing Splint + Arthrocentesis (Ringer's lactate solution) × 1 + Liquid PRF (−) × 1	Yes
Isik et al. (2023) [50]	Randomized Clinical Trial	Arthrocentesis (Saline solution) × 1 + Liquid PRF (1 mL) × 4	Yes
Isik et al. (2022) [52]	Randomized Clinical Trial	Arthrocentesis (Saline solution) × 1 + Liquid PRF (1 mL) × 4	Yes
Ghoneim et al. (2021) [53]	Randomized Clinical Trial	Arthrocentesis (Ringer's lactate solution) × 1 + Liquid PRF (1.5 mL) × 1	Yes
Karadayi et al. (2021) [54]	Randomized Clinical Trial	Arthrocentesis (Ringer's lactate solution) × 1 + Liquid PRF (2 mL) × 1	Yes
Torul et al. (2020) [55]	Randomized Clinical Trial	Arthrocentesis (Ringer's lactate solution) × 1 + Liquid PRF (1 mL) × 1	Yes
Teama et al. (2020) [56]	Randomized Clinical Trial	Superior Repositioning Splint + Arthrocentesis (Saline Solution) × 1 + Liquid PRF (1.5–2 mL) × 1	Yes

*Note*: The color shading in the table indicates yes versus no.

**TABLE 10:** Summary of findings from studies evaluating whether the adjunctive administration of liquid PRF to arthrocentesis provides any benefits in improving MMO in patients with TMD, based on comparisons between baseline and follow‐up measurements.

Authors (years)	Type of study	Experimental group (amount of injection) × Number of injection	Did the treatment provide any benefit?
Comparative clinical studies			
Tepecik et al. (2025) [40]	Retrospective Clinical Study	Arthrocentesis (Saline solution) × 1 + Liquid PRF (1.5 mL) × 1	Yes
Akkas et al. (2024) [41]	Retrospective Clinical Study	Arthrocentesis (Saline solution) × 1 + Liquid PRF (2 mL) × 1	Yes
Tepecik et al. (2024) [42]	Retrospective Clinical Study	Arthrocentesis (Saline solution) × 1 + Liquid PRF (1.5 mL) × 1	Yes
Bera et al. (2022) [43]	Retrospective Clinical Study	Arthrocentesis (Ringer's lactate solution) × 6 + Liquid PRF (2 mL) × 6	Yes
Yuce et al. (2020) [44]	Retrospective Clinical Study	Arthrocentesis (Ringer's lactate solution) × 1 + Liquid PRF (2 mL) × 3	Yes
Chaulagain et al. (2025) [46]	Randomized Clinical Trial	Arthrocentesis (Ringer's lactate solution) × 1 + Liquid PRF (2–3 mL) × 1	Yes
Nasef et al. (2024) [48]	Randomized Clinical Trial	Anterior Repositing Splint + Arthrocentesis (Ringer's lactate solution) × 1 + Liquid PRF (−) × 1	Yes
Isik et al. (2023) [50]	Randomized Clinical Trial	Arthrocentesis (Saline solution) × 1 + Liquid PRF (1 mL) × 4	Yes
Isik et al. (2022) [52]	Randomized Clinical Trial	Arthrocentesis (Saline solution) × 1 + Liquid PRF (1 mL) × 4	Yes
Ghoneim et al. (2021) [53]	Randomized Clinical Trial	Arthrocentesis (Ringer's lactate solution) × 1 + Liquid PRF (1.5 mL) × 1	Yes
Karadayi et al. (2021) [54]	Randomized Clinical Trial	Arthrocentesis (Ringer's lactate solution) × 1 + Liquid PRF (2 mL) × 1	Yes
Torul et al. (2020) [55]	Randomized Clinical Trial	Arthrocentesis (Ringer's lactate solution) × 1 + Liquid PRF (1 mL) × 1	Yes
Teama et al. (2020) [56]	Randomized Clinical Trial	Superior Repositioning Splint + Arthrocentesis (Saline Solution) × 1 + Liquid PRF (1.5–2 mL) × 1	Yes

*Note*: The color shading in the table indicates yes versus no.

*#4 Does the administration of liquid PRF as an adjunct to arthrocentesis offer additional benefit than arthrocentesis alone in reducing pain and improving MMO in patients with TMD? If so, is there a difference in outcomes between a single and multiple injections of liquid PRF following arthrocentesis?*

About 84.61% of findings from the included studies in this section showed that the administration of liquid PRF as an adjunct to arthrocentesis offered greater benefits than arthrocentesis alone in reducing pain, and 69.23% in improving MMO. The remaining findings reported no difference between these two treatment modalities (Tables 11 and 12). Only one study evaluated the outcomes of single versus multiple adjunctive injections of liquid PRF following arthrocentesis and reported no difference for both pain and MMO outcomes (Tables 13 and 14).

**TABLE 11:** Summary of findings from studies evaluating whether the adjunctive administration of liquid PRF to arthrocentesis provides more benefits than arthrocentesis alone in reducing pain in patients with TMD.

Authors (years)	Type of study	Control group (name of the solution) × Number of injection	Experimental group (amount of injection or name of the solution) × Number of injection	Which treatment was more beneficial?
Comparative clinical studies				
Tepecik et al. (2025) [40]	Retrospective Clinical Study	Arthrocentesis (Saline solution) × 1	Arthrocentesis (Saline solution) × 1 + Liquid PRF (1.5 mL) × 1	Arthrocentesis + Liquid PRF
Akkas et al. (2024) [41]	Retrospective Clinical Study	Arthrocentesis (Saline solution) × 1	Arthrocentesis (Saline solution) × 1 + Liquid PRF (2 mL) × 1	No Difference
Tepecik et al. (2024) [42]	Retrospective Clinical Study	Arthrocentesis (Saline solution) × 1	Arthrocentesis (Saline solution) × 1 + Liquid PRF (1.5 mL) × 1	Arthrocentesis + Liquid PRF
Bera et al. (2022) [43]	Retrospective Clinical Study	Arthrocentesis (Ringer's lactate solution) × 6	Arthrocentesis (Ringer's lactate solution) × 6 + Liquid PRF (2 mL) × 6	No Difference
Yuce et al. (2020) [44]	Retrospective Clinical Study	Arthrocentesis (Ringer's lactate solution) × 1	Arthrocentesis (Ringer's lactate solution) × 1 + Liquid PRF (2 mL) × 3	Arthrocentesis + Liquid PRF
Chaulagain et al. (2025) [46]	Randomized Clinical Trial	Arthrocentesis (Ringer's lactate solution) × 1	Arthrocentesis (Ringer's lactate solution) × 1 + Liquid PRF (2–3 mL) × 1	Arthrocentesis + Liquid PRF
Nasef et al. (2024) [48]	Randomized Clinical Trial	Anterior Repositing Splint + Arthrocentesis (Ringer's lactate solution) × 1	Anterior Repositing Splint + Arthrocentesis (Ringer's lactate solution) × 1 + Liquid PRF (−) × 1	Arthrocentesis + Liquid PRF
Isik et al. (2023) [50]	Randomized Clinical Trial	Arthrocentesis (Saline solution) × 1	Arthrocentesis (Saline solution) × 1 + Liquid PRF (1 mL) × 4	Arthrocentesis + Liquid PRF
Isik et al. (2022) [52]	Randomized Clinical Trial	Arthrocentesis (Saline solution) × 1	Arthrocentesis (Saline solution) × 1 + Liquid PRF (1 mL) × 4	Arthrocentesis + Liquid PRF
Ghoneim et al. (2021) [53]	Randomized Clinical Trial	Arthrocentesis (Ringer's lactate solution) × 1	Arthrocentesis (Ringer's lactate solution) × 1 + Liquid PRF (1.5 mL) × 1	Arthrocentesis + Liquid PRF
Karadayi et al. (2021) [54]	Randomized Clinical Trial	Arthrocentesis (Ringer's lactate solution) × 1	Arthrocentesis (Ringer's lactate solution) × 1 + Liquid PRF (2 mL) × 1	Arthrocentesis + Liquid PRF
Torul et al. (2020) [55]	Randomized Clinical Trial	Arthrocentesis (Ringer's lactate solution) × 1	Arthrocentesis (Ringer's lactate solution) × 1 + Liquid PRF (1 mL) × 1	Arthrocentesis + Liquid PRF
Teama et al. (2020) [56]	Randomized Clinical Trial	Superior Repositioning Splint + Arthrocentesis (Saline Solution) × 1	Superior Repositioning Splint + Arthrocentesis (Saline Solution) × 1 + Liquid PRF (1.5–2 mL) × 1	Arthrocentesis + Liquid PRF

*Note*: The color shading in the table indicates yes versus no.

**TABLE 12:** Summary of findings from studies evaluating whether the adjunctive administration of liquid PRF to arthrocentesis provides more benefits than arthrocentesis alone in improving MMO in patients with TMD.

Authors (years)	Type of study	Control group (name of the solution) × Number of injection	Experimental group (amount of injection or name of the solution) × Number of injection	Which treatment was more beneficial?
Comparative clinical studies				
Tepecik et al. (2025) [40]	Retrospective Clinical Study	Arthrocentesis (Saline solution) × 1	Arthrocentesis (Saline solution) × 1 + Liquid PRF (1.5 mL) × 1	No Difference
Akkas et al. (2024) [41]	Retrospective Clinical Study	Arthrocentesis (Saline solution) × 1	Arthrocentesis (Saline solution) × 1 + Liquid PRF (2 mL) × 1	No Difference
Tepecik et al. (2024) [42]	Retrospective Clinical Study	Arthrocentesis (Saline solution) × 1	Arthrocentesis (Saline solution) × 1 + Liquid PRF (1.5 mL) × 1	No Difference
Bera et al. (2022) [43]	Retrospective Clinical Study	Arthrocentesis (Ringer's lactate solution) × 6	Arthrocentesis (Ringer's lactate solution) × 6 + Liquid PRF (2 mL) × 6	Arthrocentesis + Liquid PRF
Yuce et al. (2020) [44]	Retrospective Clinical Study	Arthrocentesis (Ringer's lactate solution) × 1	Arthrocentesis (Ringer's lactate solution) × 1 + Liquid PRF (2 mL) × 3	Arthrocentesis + Liquid PRF
Chaulagain et al. (2025) [46]	Randomized Clinical Trial	Arthrocentesis (Ringer's lactate solution) × 1	Arthrocentesis (Ringer's lactate solution) × 1 + Liquid PRF (2–3 mL) × 1	Arthrocentesis + Liquid PRF
Nasef et al. (2024) [48]	Randomized Clinical Trial	Anterior Repositing Splint + Arthrocentesis (Ringer's lactate solution) × 1	Anterior Repositing Splint + Arthrocentesis (Ringer's lactate solution) × 1 + Liquid PRF (−) × 1	Arthrocentesis + Liquid PRF
Isik et al. (2023) [50]	Randomized Clinical Trial	Arthrocentesis (Saline solution) × 1	Arthrocentesis (Saline solution) × 1 + Liquid PRF (1 mL) × 4	Arthrocentesis + Liquid PRF
Isik et al. (2022) [52]	Randomized Clinical Trial	Arthrocentesis (Saline solution) × 1	Arthrocentesis (Saline solution) × 1 + Liquid PRF (1 mL) × 4	Arthrocentesis + Liquid PRF
Ghoneim et al. (2021) [53]	Randomized Clinical Trial	Arthrocentesis (Ringer's lactate solution) × 1	Arthrocentesis (Ringer's lactate solution) × 1 + Liquid PRF (1.5 mL) × 1	Arthrocentesis + Liquid PRF
Karadayi et al. (2021) [54]	Randomized Clinical Trial	Arthrocentesis (Ringer's lactate solution) × 1	Arthrocentesis (Ringer's lactate solution) × 1 + Liquid PRF (2 mL) × 1	No Difference
Torul et al. (2020) [55]	Randomized Clinical Trial	Arthrocentesis (Ringer's lactate solution) × 1	Arthrocentesis (Ringer's lactate solution) × 1 + Liquid PRF (1 mL) × 1	Arthrocentesis + Liquid PRF
Teama et al. (2020) [56]	Randomized Clinical Trial	Superior Repositioning Splint + Arthrocentesis (Saline Solution) × 1	Superior Repositioning Splint + Arthrocentesis (Saline Solution) × 1 + Liquid PRF (1.5–2 mL) × 1	Arthrocentesis + Liquid PRF

*Note*: The color shading in the table indicates yes versus no.

**TABLE 13:** Summary of findings from studies evaluating whether there is a difference in outcomes between single and multiple injections of liquid PRF following arthrocentesis in reducing pain in patients with TMD.

Authors (years)	Type of study	Control group (amount of injection or name of the solution) × Number of injection	Experimental group (amount of injection or name of the solution) × Number of injection	Which treatment was more beneficial?
Comparative clinical studies				
Tepecik et al. (2025) [39]	Retrospective Clinical Study	Arthrocentesis (Saline solution) × 1 + Liquid PRF (1.5 mL) × 1	Arthrocentesis (Saline solution) × 1 + Liquid PRF (1.5 mL) × 3	No Difference

*Note*: The color shading in the table indicates yes versus no.

**TABLE 14:** Summary of findings from studies evaluating whether there is a difference in outcomes between single and multiple injections of liquid PRF following arthrocentesis in improving MMO in patients with TMD.

Authors (years)	Type of study	Control group (amount of injection or name of the solution) × Number of injection	Experimental group (amount of injection or name of the solution) × Number of injection	Which treatment was more beneficial?
Comparative clinical studies				
Tepecik et al. (2025) [39]	Retrospective Clinical Study	Arthrocentesis (Saline solution) × 1 + Liquid PRF (1.5 mL) × 1	Arthrocentesis (Saline solution) × 1 + Liquid PRF (1.5 mL) × 3	No Difference

*Note*: The color shading in the table indicates yes versus no.

*#5 Does the adjunctive use of liquid PRF offer additional benefit than PRP following arthrocentesis in reducing pain and improving MMO in patients with TMD?*

About 50% of the findings showed that the adjunctive use of liquid PRF offered greater benefits than PRP in reducing pain and improving MMO in patients with TMD following arthrocentesis, while the other 50% reported no difference between these two treatment modalities (Tables 15 and 16).

**TABLE 15:** Summary of findings from studies evaluating whether the adjunctive administration of liquid PRF to arthrocentesis provides more benefits, compared with PRP administered after arthrocentesis, in reducing pain in patients with TMD.

Authors (years)	Type of study	Control group (amount of injection or name of the solution) × Number of injection	Experimental group (amount of injection or name of the solution) × Number of injection	Which treatment was more beneficial?
Comparative clinical studies				
Akkas et al. (2024) [41]	Retrospective Clinical Study	Arthrocentesis (Saline solution) × 1 + PRP (2 mL) × 1	Arthrocentesis (Saline solution) × 1 + Liquid PRF (2 mL) × 1	No Difference
Sharma et al. (2023) [51]	Randomized Clinical Trial	Arthrocentesis (Ringer's lactate solution) × 1 + PRP (2 mL) × 6	Arthrocentesis (Ringer's lactate solution) × 1 + Liquid PRF (2 mL) × 6	Arthrocentesis + Liquid PRF

*Note*: The color shading in the table indicates yes versus no.

**TABLE 16:** Summary of findings from studies evaluating whether the adjunctive administration of liquid PRF to arthrocentesis provides more benefits, compared with PRP administered after arthrocentesis, in improving MMO in patients with TMD.

Authors (years)	Type of study	Control group (amount of injection or name of the solution) × Number of injection	Experimental group (amount of injection or name of the solution) × Number of injection	Which treatment was more beneficial?
Comparative clinical studies				
Akkas et al. (2024) [41]	Retrospective Clinical Study	Arthrocentesis (Saline solution) × 1 + PRP (2 mL) × 1	Arthrocentesis (Saline solution) × 1 + Liquid PRF (2 mL) × 1	No Difference
Sharma et al. (2023) [51]	Randomized Clinical Trial	Arthrocentesis (Ringer's lactate solution) × 1 + PRP (2 mL) × 6	Arthrocentesis (Ringer's lactate solution) × 1 + Liquid PRF (2 mL) × 6	Arthrocentesis + Liquid PRF

*Note*: The color shading in the table indicates yes versus no.

*#6 Does the adjunctive use of liquid PRF offer additional benefit than HA following arthrocentesis in reducing pain and improving MMO in patients with TMD?*

50% of the findings showed that the adjunctive use of liquid PRF offered greater benefits than HA in reducing pain and improving MMO in patients with TMD following arthrocentesis, while the other 50% reported no difference between these two treatment modalities (Tables 17 and 18).

**TABLE 17:** Summary of findings from studies evaluating whether the adjunctive administration of liquid PRF to arthrocentesis provides more benefits, compared with HA administered after arthrocentesis, in reducing pain in patients with TMD.

Authors (years)	Type of study	Control group (amount of injection or name of the solution) × Number of injection	Experimental group (amount of injection or name of the solution) × Number of injection	Which treatment was more beneficial?
Comparative clinical studies				
Tepecik et al. (2025) [40]	Retrospective Clinical Study	Arthrocentesis (Saline solution) × 1 + HA (1.5 mL) × 1	Arthrocentesis (Saline solution) × 1 + Liquid PRF (1.5 mL) × 1	No Difference
Tepecik et al. (2024) [42]	Retrospective Clinical Study	Arthrocentesis (Saline solution) × 1 + HA (1.5 mL) × 1	Arthrocentesis (Saline solution) × 1 + Liquid PRF (1.5 mL) × 1	No Difference
Yuce et al. (2021) [44]	Retrospective Clinical Study	Arthrocentesis (Ringer's lactate solution) × 1 + HA (1 mL) × 1	Arthrocentesis (Ringer's lactate solution) × 1 + Liquid PRF (2 mL) × 3	Arthrocentesis + Liquid PRF
Torul et al. (2021) [55]	Randomized Clinical Trial	Arthrocentesis (Ringer's lactate solution) × 1 + HA (1 mL) × 1	Arthrocentesis (Ringer's lactate solution) × 1 + Liquid PRF (1 mL) × 1	Arthrocentesis + Liquid PRF

*Note*: The color shading in the table indicates yes versus no.

**TABLE 18:** Summary of findings from studies evaluating whether the adjunctive administration of liquid PRF to arthrocentesis provides more benefits, compared with HA administered after arthrocentesis, in improving MMO in patients with TMD.

Authors (years)	Type of study	Control group (amount of injection or name of the solution) × Number of injection	Experimental group (amount of injection or name of the solution) × Number of injection	Which treatment was more beneficial?
Comparative clinical studies				
Tepecik et al. (2025) [40]	Retrospective Clinical Study	Arthrocentesis (Saline solution) × 1 + HA (1.5 mL) × 1	Arthrocentesis (Saline solution) × 1 + Liquid PRF (1.5 mL) × 1	No Difference
Tepecik et al. (2024) [42]	Retrospective Clinical Study	Arthrocentesis (Saline solution) × 1 + HA (1.5 mL) × 1	Arthrocentesis (Saline solution) × 1 + Liquid PRF (1.5 mL) × 1	No Difference
Yuce et al. (2021) [44]	Retrospective Clinical Study	Arthrocentesis (Ringer's lactate solution) × 1 + HA (1 mL) × 1	Arthrocentesis (Ringer's lactate solution) × 1 + Liquid PRF (2 mL) × 3	Arthrocentesis + Liquid PRF
Torul et al. (2021) [55]	Randomized Clinical Trial	Arthrocentesis (Ringer's lactate solution) × 1 + HA (1 mL) × 1	Arthrocentesis (Ringer's lactate solution) × 1 + Liquid PRF (1 mL) × 1	Arthrocentesis + Liquid PRF

*Note*: The color shading in the table indicates yes versus no.

## 4 DISCUSSION

In this narrative review, we comprehensively assessed whether liquid PRF, either as a standalone therapy or as an adjunct to other treatment modalities, offered any additional clinical benefit for patients with TMD.

**FIGURE 3:**
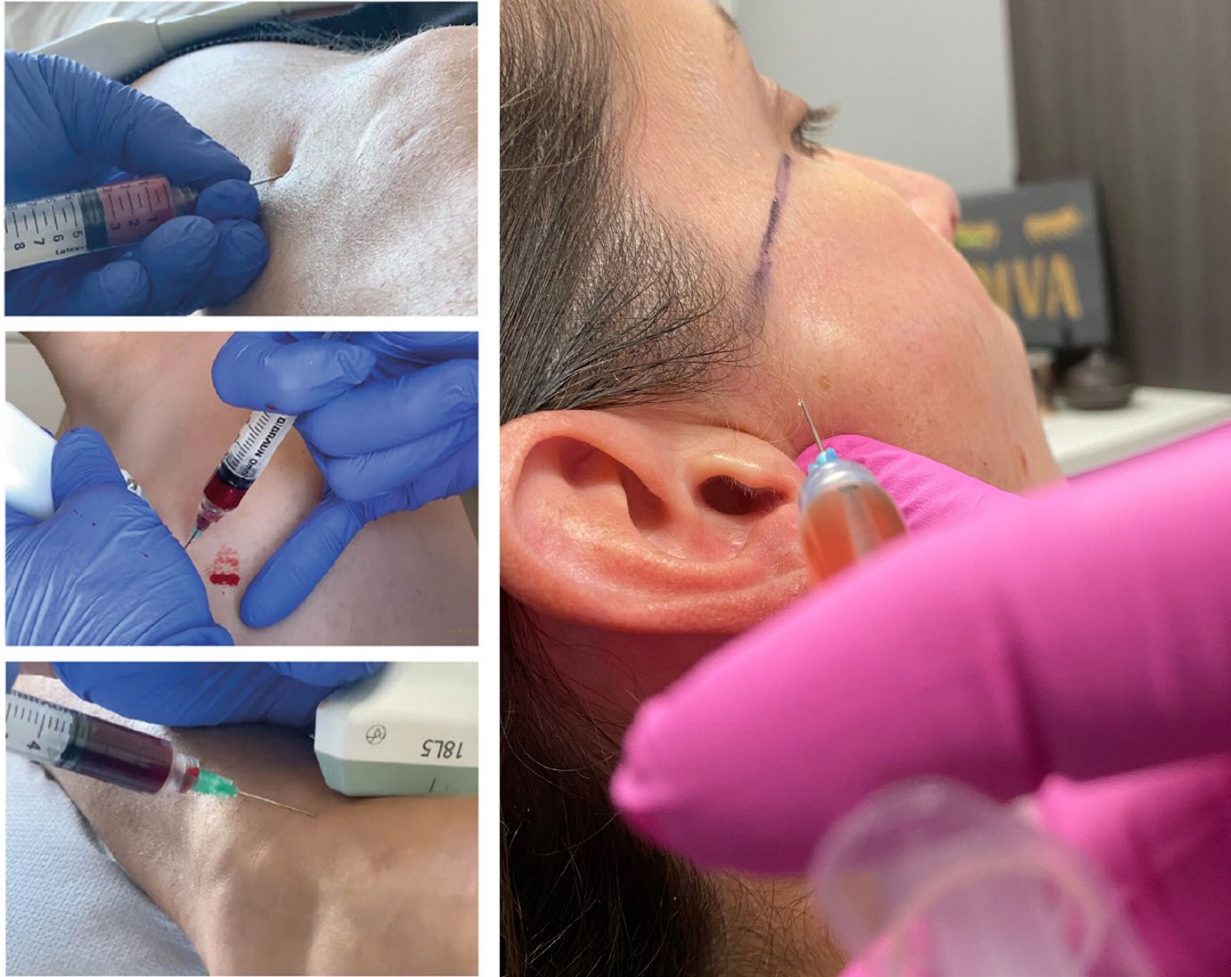
Injections of liquid PRF into joints or other anatomical spaces. Reprinted with permission from Miron et al.^64^

Liquid PRF was first introduced in 2015 as an injectable formulation of PRF that remains in a liquid state for ~ 15–20 min.^26^, ^27^ During this period, fibrinogen has not yet converted into a fibrin matrix, allowing it to be easily injected into joints or other anatomical spaces before clotting occurs (Figure 3; see QR Codes 1 and 2 for visual demonstrations).^26^, ^63^, ^64^ In 2017, the low‐speed centrifugation concept (LSCC) was further developed to enhance the regenerative potential of liquid PRF by reducing both the centrifugation speed and time on a fixed‐angle centrifugation device, which allowed for a liquid formulation of PRF.^65^ Most of the studies included in this review utilized these original protocols (700 rpm (60 g) for 3 min) for the preparation of liquid PRF. However, further advancements to protocols have since been established, which have been discussed in future sections.

**Figure:**
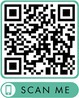
**QR Code 1**. TMJ injection using liquid PRF.

**Figure:**
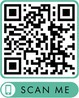
**QR Code 2**. Orthopedic joint therapy using liquid PRF.

Across the included studies, the administration of liquid PRF has been shown to have beneficial effects in reducing pain and enhancing MMO in patients with TMD. In the majority of studies, the adjunctive use of liquid PRF following arthrocentesis, the most commonly used minimally invasive treatment modality for TMD, demonstrated greater positive effects compared with arthrocentesis alone in reducing pain, improving MMO, or both.^40^, ^42^, ^44^, ^46^, ^48^, ^50^, ^52^, ^53^, ^54^, ^55^, ^56^ Similarly, based on the results of three randomized clinical trials (RCTs) within a recent scoping review, it was reported that articular pain decreased to 0–25% of initial preinterventional values at 3 months in the treatment groups (arthrocentesis + liquid PRF), compared with 38–50% in the control groups (arthrocentesis alone).^37^ Mandible mobility also showed greater improvement in the treatment groups, increasing to 121–153% versus 115–120% in the controls.^37^ These enhanced effects can be attributed to the biological properties of liquid PRF, which contains a variety of growth factors, including platelet‐derived growth factor (PDGF), insulin‐like growth factor (IGF), vascular endothelial growth factor (VEGF), and transforming growth factor beta‐1 (TGF‐β1), all of which can promote regeneration at both the cellular and tissue levels,^29^ including chondrocytes and cartilage tissue.^66^ Furthermore, PRF has been shown to modulate the inflammatory response by promoting the polarization of macrophages from the pro‐inflammatory M1 phenotype to the anti‐inflammatory M2 phenotype.^67^ Additionally, it has been suggested that leukocytes embedded within the PRF fibrin matrix may contribute to anti‐nociceptive effects by releasing anti‐inflammatory cytokines, chemokines, and opioid peptides.^68^

The data on whether multiple injections of liquid PRF following arthrocentesis are more beneficial than a single injection were very limited, with only one study investigating this.^39^ However, that study found no difference between single and multiple injections.^39^ While the literature generally suggests that multiple injections are often more effective due to their cumulative physiological effect,^69^, ^70^, ^71^, ^72^ some studies have reported no significant differences.^73^, ^74^, ^75^ Given the limited available data, no definitive conclusions can be drawn, which further highlights the need for additional research.

In terms of comparing the adjunctive effects of liquid PRF and PRP following arthrocentesis, only two studies were included.^41^, ^51^ One demonstrated superior clinical outcomes with liquid PRF,^51^ while the other reported no difference between the two in reducing pain or improving MMO.^41^ However, it is important to note that the former study involved six injections of liquid PRF or PRP,^51^ while the latter administered single injections.^41^ Interestingly, the latter study also showed that PRP provided better improvement in MMO during the early postoperative period, whereas liquid PRF was more effective in the later stages.^41^ This has been demonstrated in several studies, where PRP is associated with a rapid burst release of growth factors, while liquid PRF provides a more prolonged and sustained release.^28^, ^76^ A recent systematic review also reported that liquid PRF was favored over PRP in 72% of studies across various disciplines, while 24% found no significant differences between the two.^28^

In terms of comparing the adjunctive effect of liquid PRF and HA following arthrocentesis, four studies were included.^40^, ^42^, ^44^, ^55^ Two demonstrated superior clinical outcomes with liquid PRF,^44^, ^55^ while the other two reported no difference between the two in reducing pain or improving MMO.^40^, ^42^ It is also important to note that in one of the studies reporting greater benefit with liquid PRF, the PRF was administered three times at a higher volume (2 mL), whereas HA was injected only once and at a lower volume (1 mL),^44^ which may have influenced the observed outcomes. While both agents exhibit anti‐inflammatory properties,^77^ their mechanisms of action differ. HA has been suggested to exert its effects by enhancing nourishment, lubrication, and maintaining joint stability.^36^ In contrast, liquid PRF exerts its effects through the sustained release of growth factors and cytokines over time.^8^ Taken together, these findings suggest that liquid PRF could be a viable alternative to HA following arthrocentesis, with the potential to offer comparable or even superior clinical outcomes.

Corroborating our results, a previous systematic review and network meta‐analysis also compared the effectiveness of HA, PRP, and PRF for TMD.^36^ It was reported that PRP and PRF had comparable short‐term efficacy, but PRF was more advantageous in the long term.^36^ Despite this, their results warrant cautious interpretation due to imprecision and inconsistency based on the GRADE system. Furthermore, they grouped the injections of HA, PRP, or PRF (either alone or with arthrocentesis) together and compared them to arthrocentesis alone, rather than isolating the effects of these biologics when used independently or as adjuncts.^36^

Assessing potential adverse effects is an important aspect of evaluating any treatment. Therefore, it is worth mentioning that only three studies assessed the adverse effects of treatment, and none reported any.^52^, ^59^, ^62^ As liquid PRF is an autologous platelet‐derived biomaterial, it is generally regarded as a safe treatment option, with minimal risk of complications due to the low likelihood of immune reactions.^63^

## 5 OPTIMIZATION OF LIQUID PRF

One noteworthy observation was that many studies utilized older centrifugation protocols to produce liquid PRF. Within the past 5 years, many research studies have demonstrated an ability to further optimize PRF by concentrating liquid PRF over 10‐fold by utilizing more novel centrifugation protocols.

**FIGURE 4:**
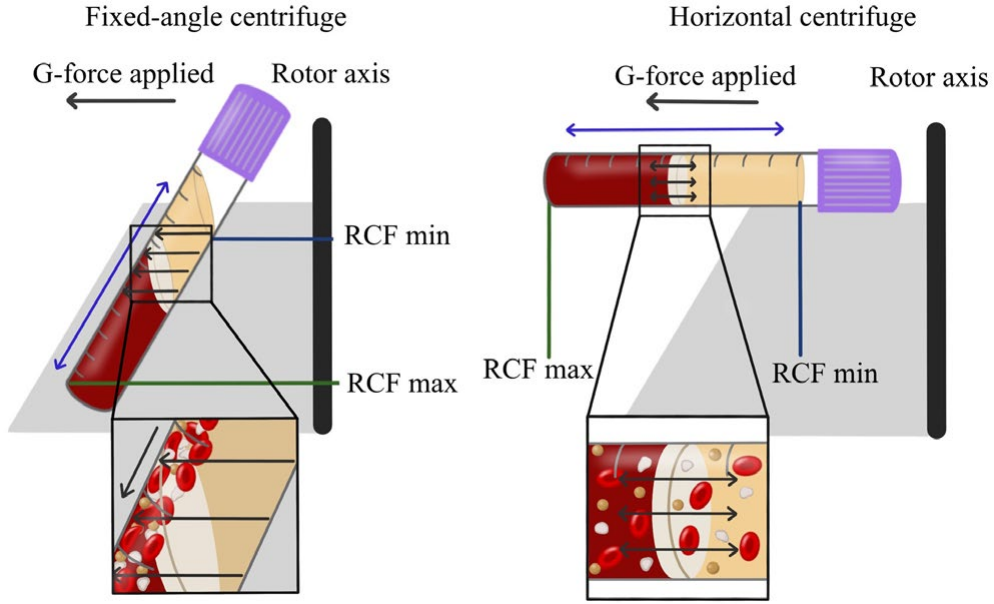
Illustrations comparing fixed‐angle and horizontal centrifuges. Following centrifugation on fixed‐angle centrifuges, blood layers do not separate evenly and, as a result, an angled blood separation is observed. In contrast, horizontal centrifugation produces an even separation. Owing to the large RCF values (~200–700 g), cells are pushed toward the outside and downwards. On a fixed‐angle centrifuge, cells are pushed toward the back of centrifugation tubes and then downwards/upwards based on cell density. These g‐forces produce additional shear stress on cells as they separate based on density along the back walls of centrifugation tubes. In contrast, horizontal centrifugation allows for the free mobility of cells to separate into their appropriate layers based on density, allowing for more optimal cell separation as well as less trauma/shear stress on cells. Reprinted with permission from Miron et al. 2019.^78^

Some major breakthroughs were made in 2019 when it was discovered that horizontal centrifugation of PRF led to a 4‐fold increase in platelet and leukocyte concentrations when compared with fixed‐angle devices.^78^, ^79^ Not only was PRF more concentrated with cells, but by not driving the cells against the back walls during fixed‐angle centrifugation, the PRF remains liquid much longer (clotting always starts from the tube wall surfaces; Figure 4).^80^

**FIGURE 5:**
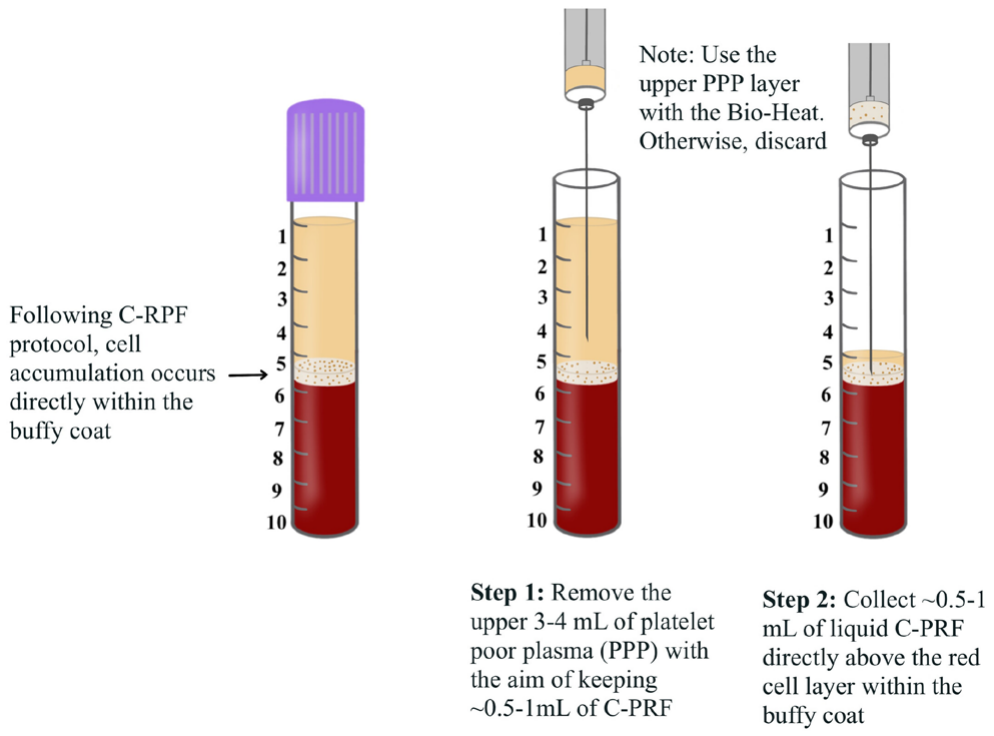
Method to collect and concentrate C‐PRF. Following centrifugation at higher speeds (2000 g for 8 min), the majority of cells are located directly at the buffy coat layer. Instead of attempting to remove this layer with a long needle into the deep layers, it is highly advised to first remove the upper 4 mL layer of platelet‐poor plasma (PPP), followed by collection of the C‐PRF buffy coat layer. Reprinted with permission from Miron et al.^80^

The final concentration of platelets and leukocytes in the original liquid PRF protocols (700 rpm for 3 min) only represented a 207% and 123% increase, respectively, and only 27% and 16% of the total platelets and leukocytes, respectively.^78^ As a result, future research focused on better concentrating the cells at the buffy coat zone utilizing higher centrifugation protocols. Because of the advantages of horizontal centrifugation, it was possible to concentrate, whereas a 1200–1700% increase in platelet numbers could easily be achieved by harvesting this 0.3–0.5 mL of concentrated platelet‐rich (C‐PRF; total platelet concentrations of >2000–3000 × 10^9^ cells/L).^81^, ^82^ These protocols selectively concentrate these cells within a thin layer between the plasma and red blood cell interface (buffy coat zone; Figure 5).

Lastly, the importance of PRF tubes cannot be overstated. Many tubes have been found with various chemical additives such as silicone and silica.^83^ It has previously been reported that such additional substances may increase the likelihood of additional inflammation, which may lead to additional pain reported by the patients.^83^ This may be one simple reason for the variability observed in the reported studies in this article. Furthermore, by modifying PET tubes to create liquid PRF by making them more hydrophilic and by utilizing the Bio‐Cool technology, it is possible to keep liquid PRF liquid for up to 4 h.^84^

## 6 LIMITATIONS AND FUTURE PROSPECTS

To provide a comprehensive review, we included all available comparative and non‐comparative evidence, such as RCTs and non‐RCTs. However, this approach may have introduced a limitation, as including non‐RCT comparative studies as well as non‐comparative studies without control groups increases the risk of bias and limits the ability to draw strong, evidence‐based conclusions due to the lack of proper randomization, robust methodology, etc. While these studies can offer valuable insights, caution is necessary when interpreting the results. Furthermore, although our review did not follow the strict guidelines of a systematic review, we applied a systematic search strategy, carefully selecting and extracting the data. Gray literature was also considered to reduce the risk of missing relevant studies; however, our search primarily relied on Google Scholar, which lacks clear indexing criteria and may have provided more limited coverage than specialized gray literature databases or repositories, which were not included in our study. Additionally, the quality and risk of bias of the included studies were not assessed; therefore, all data should be interpreted with caution.

Additionally, the wide range of variables and confounding factors across the included studies hindered a reliable quantitative synthesis, as only a limited number of studies shared identical parameters. These differences included variations in study design, TMD types and subtypes, treatment and control groups, the volume of solution used for arthrocentesis, the volume of injected liquid PRF and other biologics, the frequency and intervals of their injections, and the duration of follow‐up periods. Therefore, future research should focus on standardizing protocols to minimize variability and confounding factors, enabling more robust analyses that can integrate findings across multiple studies.

We also did not apply any restrictions regarding age, gender, or smoking status for study inclusion or exclusion, and none of the included studies assessed these sociodemographic factors as determinants of treatment outcomes. Nevertheless, a notable observation in our study was that most patients in the studies included in this review were female, which aligns with epidemiological research consistently showing that TMD is 2–5 times more common in females than in males.^85^, ^86^ It has also been reported that TMD is most prevalent among individuals of a younger age.^87^ Additionally, a study demonstrated that current smokers had three times the odds of developing TMD compared with nonsmokers.^88^ Smokers with TMD have also been shown to report greater pain severity than nonsmokers, placing them at higher risk for factors that may negatively influence treatment outcomes.^89^ Therefore, future studies should investigate whether factors such as gender, age, or smoking status influence the clinical effectiveness of liquid PRF in the treatment of TMD.

Future research could also explore the potential of innovative approaches to enhance the outcomes of liquid PRF for TMD treatment. For instance, none of the included studies used horizontal centrifugation devices to produce liquid PRF. Horizontal centrifugation, which offers more uniform cell distribution and higher cell concentration compared with conventional fixed‐angle centrifugation devices,^79^, ^90^ could be investigated for its efficacy in optimizing PRF matrices in this context. Furthermore, the novel C‐PRF protocols could provide additional clinical improvements. This enhanced cellular concentration may offer further advantages in future studies exploring liquid PRF applications.^80^, ^81^, ^82^

## 7 CLINICAL RECOMMENDATIONS

**FIGURE 6:**
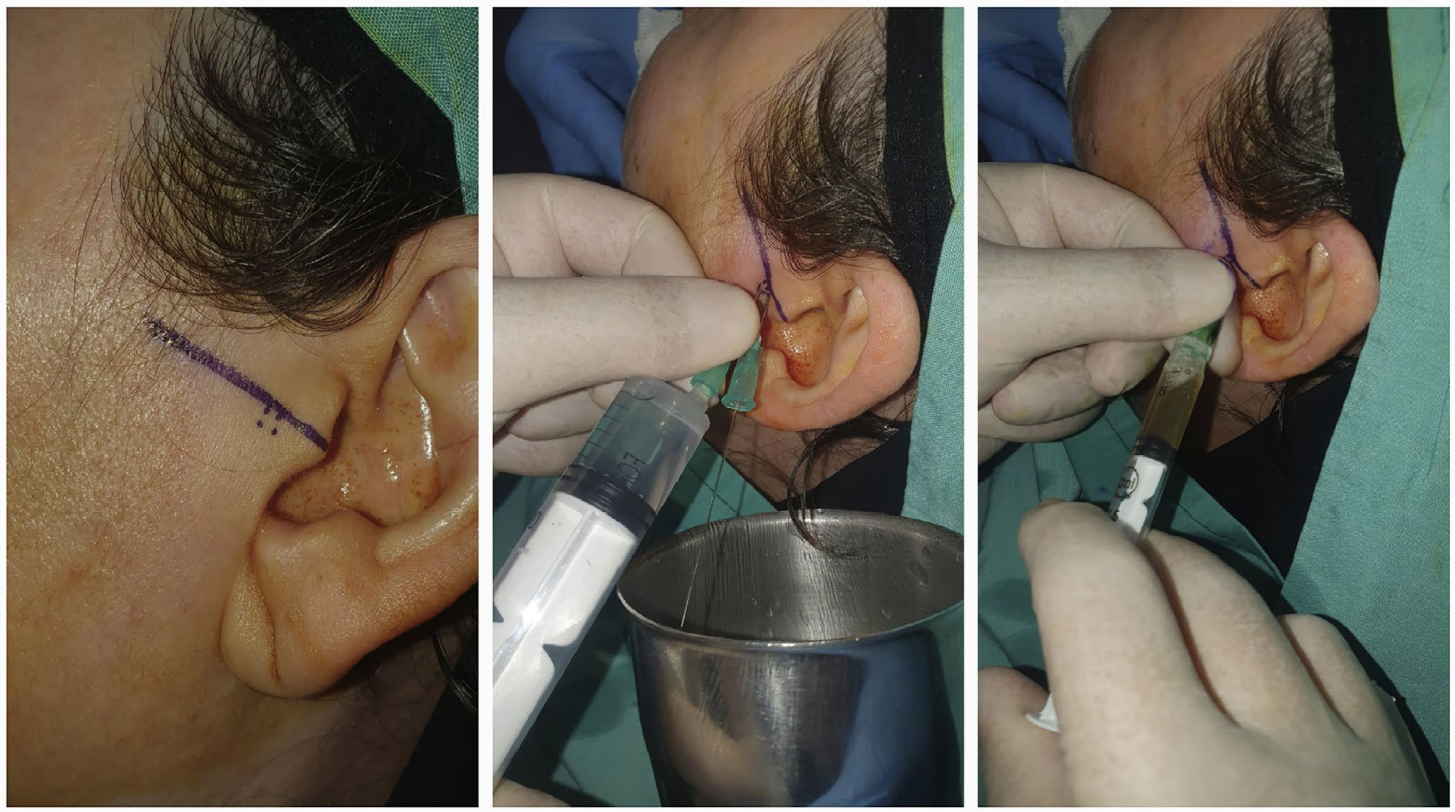
Arthrocentesis procedure and post‐procedural liquid PRF injection. Reprinted with permission from Akkas et al.^41^

Based on the current evidence, the injection of liquid PRF following arthrocentesis has been most commonly used in the included studies and has shown favorable results compared with arthrocentesis alone. It has also demonstrated equal or superior outcomes when compared with alternative injectables such as PRP or HA following arthrocentesis. It has previously been reported that this combination approach first achieves mechanical relief and lavage of the joint space through arthrocentesis, followed by enhanced healing/regeneration in combination with reduced inflammation via the injection of liquid PRF (Figure 6).^41^ This sequential protocol seems to synergize both mechanical and biological benefits and leads to better clinical outcomes for the management of TMD. Clinicians may consider the following step‐by‐step protocol to utilize this technique effectively.

1. *Local Anesthesia*: The auriculotemporal nerve should be anesthetized by infiltrating 2% lidocaine with epinephrine (1:100 000).
2. *Preparation of Liquid PRF*: Collect 40 mL of venous blood into four sterile PET plastic 10 mL anticoagulant‐free tubes. Then, centrifuge the blood immediately using a horizontal centrifugation system (BioPRF Centrifuge, USA) at 2000 g for 8 min. Following the centrifugation process, discard the upper 3–4 mL of platelet‐poor plasma (PPP), collect 0.5–1 mL of the liquid fraction containing the buffy coat layer (C‐PRF) into each syringe (see QR Code 3 for a visual demonstration). Place the tubes/syringes in a cooling device (Bio‐Cool Device, USA) to delay the clotting process and help maintain the liquid PRF in its liquid state for a longer period (the conversion of fibrinogen + thrombin into a fibrin clot is an enzymatic process and enzymes do not function as optimally at colder temperatures).^84^

**Figure:**
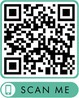
**QR Code 3**. Drawing concentrated PRF (C‐PRF).

- It should also be noted that different protocols can be applied for the injection of liquid PRF. However, as we previously mentioned, C‐PRF contains higher amounts of platelets and leukocytes; therefore, we have recommended its use in our step‐by‐step protocol.

3 *Arthrocentesis*: Begin by thoroughly disinfecting the preauricular area and draping the site using a sterile technique. Identify the anatomical landmarks by drawing a line from the tragus of the ear to the lateral canthus of the eye. Mark two points along this line: the first point, located 10 mm anterior to the tragus and 2 mm below the line, which serves as the posterior entry point. The second point, 20 mm anterior to the tragus and 10 mm below the line, serves as the anterior exit point. Both points correspond to access sites within the superior joint space of the TMJ. Insert a 20‐gauge needle at the posterior point to access the joint, and inject 2–3 mL of Ringer's lactate or sterile saline to distend the superior compartment. Then, insert a second needle at the anterior point to establish an outflow tract. Proceed with gentle lavage using the same solution, instilling ~ 100–150 mL under low pressure to flush out inflammatory mediators, debris, and to release intra‐articular adhesions.
4 *Injection of Liquid PRF*: After completing the lavage, remove the anterior needle while leaving the posterior needle in place. Through the remaining posterior needle, slowly inject 1–2 mL of liquid C‐PRF into the superior joint space. After the injection is completed, carefully withdraw the needle and apply gentle pressure to the site to minimize bleeding and discomfort.
5 *Post‐procedure Instructions*: After the procedure, apply a cold compress to the treated area to help minimize swelling. Advise the patient to follow a soft diet for the next few days and to avoid excessive mouth opening during this period. Starting a few days posttreatment, recommend physiotherapy or gentle jaw mobility exercises to support functional recovery. Analgesics, such as ibuprofen or acetaminophen, may also be used as needed to manage discomfort.

## 8 CONCLUSION

Based on the current evidence, the administration of liquid PRF has been shown to have beneficial effects in reducing pain and enhancing MMO in patients with TMD. Most studies also reported that the adjunctive use of liquid PRF following arthrocentesis resulted in more positive effects compared with arthrocentesis alone. When used adjunctively with arthrocentesis, liquid PRF showed comparable or superior clinical outcomes in terms of pain and MMO compared with PRP and HA. Therefore, liquid PRF could act as a viable alternative to both PRP and HA, potentially offering comparable or even superior clinical outcomes. Nevertheless, these results should be interpreted with caution due to the high variability among the included studies. Well‐designed, high‐quality RCTs with standardized methodologies are further needed to facilitate quantitative analyses that can effectively integrate and compare findings from multiple investigations.

## AUTHOR CONTRIBUTIONS

All authors made substantial contributions to the conception and design of the manuscript. NF and MA performed the literature search. All authors drafted the work and revised it critically for important intellectual content, agreed to be accountable for all aspects of the study design and its content, and approved the final submitted version.

## CONFLICT OF INTEREST STATEMENT

Richard J Miron is the founder of Miron Research and Development in Dentistry LLC, which holds intellectual property on the production of PRF. All other authors declare that they have no competing interests.

## ETHICS STATEMENT

No ethics approval was required for this study since it was a narrative review.

## INFORMED CONSENT

No informed consent was required.

## Data Availability

The datasets used and/or analyzed during the current study are available from the corresponding author upon reasonable request.
